# Impact of Seven-Day Versus Five-Day Inpatient Physiotherapy: A Review of Clinical and Economic Outcomes

**DOI:** 10.7759/cureus.108221

**Published:** 2026-05-04

**Authors:** Juan Elicio Hernandez Xumet, Josmarlin Gonzalez Perez, Juan Claudio Garcia Thompson, Jeronimo Pedro Fernandez Gonzalez

**Affiliations:** 1 Physical Medicine and Pharmacology, Universidad de La Laguna, Santa Cruz de Tenerife, ESP

**Keywords:** after-hours care, cost-benefit analysis, health equity, inpatient physiotherapy, length of stay, subacute rehabilitation, weekend physiotherapy

## Abstract

Inpatient physiotherapy is traditionally delivered on a five-day (Monday to Friday) schedule. Although prolonged bed rest leads to rapid physiological deconditioning, the clinical and economic justification for comprehensive seven-day physiotherapy remains contested, with notable disparities in implementation across hospital departments. This narrative review, with systematic components, synthesises evidence on the clinical effectiveness, economic viability, and implementation challenges of seven-day versus five-day physiotherapy models across various hospital specialties. Twenty-three studies, including randomised controlled trials, large-scale cohort studies, and systematic reviews, were analysed to evaluate outcomes such as hospital length of stay, functional independence, and cost-effectiveness (incremental cost per quality-adjusted life year). The evidence indicates that seven-day physiotherapy provides consistent, high-value benefits in specific settings. In subacute rehabilitation, weekend therapy reduces length of stay by approximately 2.35 days and is highly cost-effective, saving over 41,000 Australian dollars per quality-adjusted life year. Among post-hip fracture and intensive care populations, continuous therapy accelerates discharge readiness, improves survival, and reduces severe deconditioning, with a clear dose-response relationship between therapy frequency and functional recovery.

In contrast, in acute general medical and surgical wards, data from large stepped-wedge cluster trials show that routine weekend physiotherapy does not provide consistent clinical benefit, making universal coverage in these settings economically unjustified. Effective implementation requires addressing staffing skill mix, staff work-life balance, and structural inequities that limit access for vulnerable populations. Healthcare systems should therefore prioritise the implementation and funding of seven-day physiotherapy in subacute rehabilitation, acute stroke units, post-hip fracture pathways, and intensive care units while adopting targeted, criteria-based interventions in acute general internal medicine wards.

## Introduction and background

Background and rationale

Physiotherapy services in acute hospital settings have traditionally been provided from Monday to Friday, mirroring broader healthcare staffing and resource allocation patterns, with minimal or no weekend coverage [1-3]. Recent evidence indicates that the timing, frequency, and continuity of physiotherapy interventions can substantially affect patient outcomes across various domains [4-6].

**Figure 1:**
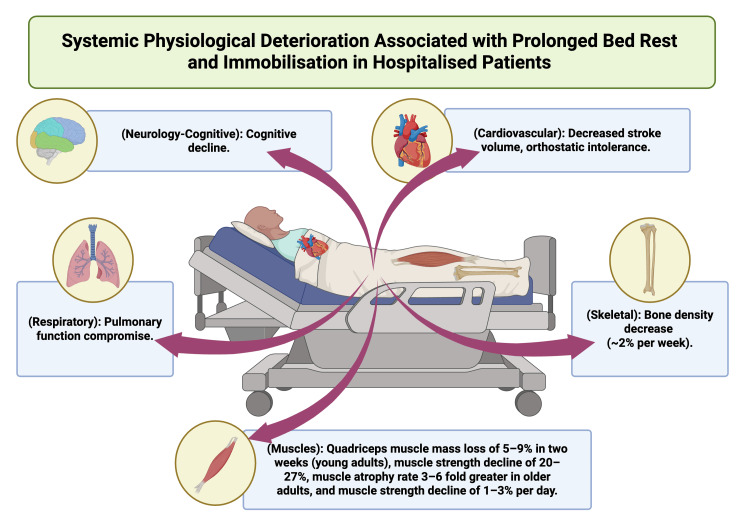
The clinical problem: systemic physiological deterioration associated with prolonged bed rest and immobilisation in hospitalised patients This figure illustrates the multi-system impact of inactivity. Labels detail quantitative decline: Muscles: quadriceps muscle mass loss of 5-9% in two weeks (young adults), muscle strength decline of 20-27%, muscle atrophy rate 3-6-fold greater in older adults, and muscle strength decline of 1-3% per day. Cardiovascular: decreased stroke volume, orthostatic intolerance. Skeletal: bone density decrease (~2% per week). Respiratory: pulmonary function compromise. Neurology-cognitive: cognitive decline. This figure was created using BioRender.

Hospitalised patients, especially those who are critically ill or recovering from acute medical or surgical conditions, undergo rapid physiological deterioration during periods of immobilisation and bed rest [7-9]. In the first two weeks of immobilisation, healthy young adults lose 5-9% of quadriceps muscle mass and 20-27% of muscle strength, while older adults experience atrophy rates three to six times higher [7]. Bed rest negatively impacts nearly every organ system, resulting in decreased stroke volume, orthostatic intolerance, impaired respiratory function, cognitive decline, and an approximate 2% weekly reduction in bone density [7,8,10]. These adverse effects, together with current pressures to shorten hospital stays, highlight the clinical necessity of minimising interruptions in physiotherapy provision and have driven the adoption of comprehensive seven-day healthcare models [1-3,5] (see Figure 1).

Mechanisms underlying physiotherapy effectiveness and dose-response relationships

The effectiveness of continuous seven-day physiotherapy is supported by several interrelated mechanisms, including physiological, neurological, psychological, and contextual factors. From a physiological standpoint, early mobilisation mitigates rapid muscle atrophy associated with bed rest, during which muscle strength typically declines by approximately 1-3% per day [10,11]. Physical therapy further enhances cardiovascular and pulmonary function, reduces thromboembolic risk, and is associated with shorter hospital and intensive care unit (ICU) stays [6-8,12,13].

From a neurological perspective, ICU-acquired weakness, which affects up to one-third of critically ill patients, and critical illness polyneuropathy or myopathy are driven by complex pathophysiological mechanisms such as microcirculatory dysfunction, mitochondrial impairment, altered membrane excitability, and axonal degeneration [14-18].

Early physical activity can mitigate these effects and promote neuroplasticity through angiogenesis and the release of neurotrophic factors, potentially reducing delirium and improving cognitive outcomes [19,20]. Research on neuroplasticity further demonstrates that higher doses of motor training, particularly through increased repetition density and temporal compression rather than cumulative minutes alone, are significant drivers of neuroplastic adaptation [21-23]. Consistent with these findings, dose-response data indicate that increased therapy frequency is independently associated with functional improvement and successful discharge home in large inpatient cohorts [24,25].

Psychological mechanisms also play a significant role in physiotherapy outcomes. Factors such as patient motivation, sense of coherence, and depressive symptoms substantially influence functional recovery, independent of disease-specific variables [26]. The incorporation of psychological and cognitive-behavioral strategies by physiotherapists, including goal setting, graded activity, and motivational techniques, can enhance patient engagement and improve outcomes. Nevertheless, the application of these approaches is frequently constrained by limited time and competing clinical demands [27].

Contextual and sociological factors shape the mechanistic pathways and influence the practical impact of seven-day physiotherapy services. Health equity and social determinants of health, including rural residence and socioeconomic status, contribute to substantial disparities in rehabilitation delivery and access to intensive inpatient physiotherapy [28]. Although weekend physiotherapy may provide benefits such as improved patient flow and reduced adverse events like falls, these must be weighed against contextual challenges. Such challenges include staffing with less experienced personnel, inefficiencies from double-handling, and hidden costs related to service coordination [1,2,28-30].

From a health systems perspective, the implementation of a seven-day physiotherapy model is primarily driven by the potential for cost savings through reduced hospital length of stay (LOS). Preliminary evidence suggests that weekend allied health services may be cost-effective in subacute rehabilitation wards. However, the cost-effectiveness of these services in acute general medical and surgical wards remains highly uncertain [3,31]. Hospital administrators must balance anticipated bed-day savings against substantial direct and indirect costs, including weekend penalty rates, the employment of less experienced rotational staff, and inefficiencies related to double-handling and service coordination [1,2]. Accurately assessing the cost-effectiveness and opportunity costs of weekend physiotherapy has therefore become a critical consideration for resource allocation.

Current evidence, gaps, and review objectives

The evidence base for seven-day physiotherapy demonstrates substantial variation across hospital specialties. The literature indicates clear benefits in subacute and post-acute orthopaedic populations, where additional weekend therapy is associated with reduced LOS and improved functional and discharge outcomes [1,4,5,32,33]. National audits report that each additional day of physiotherapy during the first postoperative week following hip fracture increases the likelihood of discharge and results in significant bed-day savings. Meta-analyses further show that supplementary physiotherapy can reduce LOS by approximately three days in subacute settings [6,33]. However, the effectiveness and cost-effectiveness of weekend physiotherapy services in acute general medical and surgical wards remain uncertain, constrained by methodological heterogeneity, variable intervention characteristics, and diverse patient populations [3,6].

Although seven-day physiotherapy services are increasingly implemented, the specific mechanistic pathways contributing to observed outcomes remain insufficiently understood, and optimal service delivery models have yet to be clearly defined. Comprehensive analysis is required to address implementation barriers, context-specific cost-effectiveness, and differential effects across clinical populations [1,2]. This narrative review aims to synthesise current evidence regarding the impact and effectiveness of comprehensive seven-day physiotherapy compared with five-day models across various hospital specialties. Through systematic analysis of physiological, neurological, psychological, and contextual mechanisms, as well as methodological approaches and outcome measures, this review seeks to develop an integrated conceptual framework to inform hospital service planning, optimise patient selection criteria, and identify priorities for future research.

## Review

Methods

Search Strategy and Study Selection

A comprehensive literature search was undertaken across multiple databases, including PubMed (MEDLINE), Cumulative Index to Nursing and Allied Health Literature (CINAHL), Physiotherapy Evidence Database (PEDro), and the Cochrane Library. To enhance reproducibility, the search strategy incorporated both Medical Subject Headings (MeSH) and free-text keywords, combining three concept blocks using Boolean operators: (1) intervention terms (e.g., "weekend" OR "seven-day" OR "after-hours" OR "increased frequency"), (2) setting terms (e.g., "inpatient" OR "acute care" OR "rehabilitation"), and (3) profession terms (e.g., "physiotherapy" OR "physical therapy" OR "allied health"). To maximise literature coverage, reference lists of included studies and relevant systematic reviews were also hand-searched to identify additional eligible publications.

Studies were included if they met the following criteria: (1) comparison of comprehensive physiotherapy services delivered six or seven days per week with standard care models provided five days per week (Monday to Friday); (2) conduct within acute or subacute hospital inpatient settings; (3) reporting of at least one relevant primary or secondary outcome, such as LOS, functional independence, discharge destination, quality of life, readmission rates, adverse events, or cost-effectiveness; and (4) publication in English between January 2000 and December 2025. Studies were excluded if they focused exclusively on outpatient or community-based rehabilitation services or paediatric populations or lacked sufficient methodological detail for robust quality assessment.

To address the systematic components of this narrative review, two independent reviewers screened titles and abstracts against predefined eligibility criteria using standardised forms. Full-text articles of potentially eligible studies were then independently assessed, and any disagreements were resolved through discussion and consensus.

Data Extraction and Quality Assessment

Data extraction utilised a standardised template to record study design, clinical setting, sample size, patient characteristics, intervention parameters (frequency, duration, intensity, and content), comparators, outcome measures, and principal findings. To address the diversity of clinical environments, studies were prospectively categorised by hospital specialty: subacute physiotherapy or rehabilitation, acute neurotreatment or stroke, post-hip fracture care, orthopaedic arthroplasty, and acute general medical or surgical wards.

Methodological quality and risk of bias were assessed using tools appropriate for each study design: the Cochrane Risk of Bias tool (RoB 2) for randomised controlled trials (RCTs) and the Newcastle-Ottawa Scale (NOS) for observational and cohort studies. Quality appraisal informed the interpretation of findings; however, studies were not excluded solely on the basis of risk of bias. Given anticipated heterogeneity in study designs, populations, and outcome metrics, a narrative synthesis approach was adopted rather than a quantitative meta-analysis.

Outcome Measures

Outcomes were categorised into operational, clinical, and economic domains. Primary operational and clinical outcomes included hospital LOS, functional independence (measured using validated tools such as the Functional Independence Measure (FIM), Barthel Index, or Activity Measure for Post-Acute Care (AM-PAC)), and discharge destination. Secondary outcomes included health-related quality of life (e.g., EQ-5D scores), 30-day readmission rates, mobility milestones, and adverse event incidence. Economic outcomes focused on cost-effectiveness, including incremental cost per quality-adjusted life year (QALY) and bed-day savings.

Results

Overview of the Included Studies

A total of 23 studies met the inclusion criteria, including 10 RCTs, eight quasi-experimental or prospective cohort studies, and five systematic reviews or meta-analyses [3-6,24,31,32,34]. Sample sizes ranged from 130 participants in single-centre quasi-experimental studies to 243,779 participants in large retrospective cohort analyses [24]. Treatment durations generally spanned the entire inpatient stay, with intervention periods ranging from six to 12 months and follow-up extending up to 12 months post-discharge [5,6,35]. Methodological quality was moderate to high in randomised trials, with most scoring 7-8 out of 10 on the PEDro scale [36].

Subacute Physiotherapy or Rehabilitation

The most robust evidence for extended physiotherapy comes from subacute rehabilitation settings. Earlier trials indicated a trend toward shorter LOS without evidence of harm [35]. A landmark RCT involving 996 patients at two facilities demonstrated that six-day physiotherapy (Monday to Saturday), compared to five-day standard care, resulted in greater functional independence (FIM improvement: mean difference 2.1 points, 95% CI: 0.4-3.8) and higher health-related quality of life at discharge. There was also a trend toward a two-day reduction in LOS (95% CI: 0-4; p=0.10) [34,37]. A subsequent meta-analysis confirmed that additional weekend allied health services in subacute rehabilitation wards significantly reduced hospital LOS by 2.35 days (95% CI: 0.45-4.24; I²=0%) [3].

Economic evaluations embedded alongside these RCTs showed that Saturday physiotherapy was cost-saving. The intervention saved AUD$41,825 per QALY gained and AUD$16,003 per minimal clinically important difference in functional independence, with a 96% probability of cost-effectiveness at a zero willingness-to-pay threshold, and these savings were sustained at 12 months post-discharge [38,39]. In mixed inpatient treatment, a pragmatic implementation of six-day physiotherapy showed a trend toward a 1.7‑day reduction in LOS and significantly greater gains in balance and functional independence among highly dependent patients [4].

Acute Neurotreatment and Stroke

In acute neurorehabilitation, a prospective cohort study of 536 patients demonstrated that implementation of a seven-day neurophysiotherapy service increased the proportion of patients assessed within 24 hours from 67% to 91%, reduced physiotherapy LOS by 7.4 days (95% CI: 3.9-10.9), and reduced total hospital LOS by 8.6 days (95% CI: 4.9-12.3) [5]. Carers strongly preferred the seven-day service, whereas patients often placed a higher value on continuity with the same therapist [5].

A meta-analysis of individual patient data from stroke rehabilitation trials found that additional weekend therapy reduced LOS by 7.5 days after adjustment for confounders, but did not significantly improve walking speed or health-related quality of life at discharge, suggesting that weekend therapy accelerates discharge readiness rather than altering the ultimate functional plateau [32].

Post-hip Fracture Care and Orthopaedic Arthroplasty

Large-scale observational data from the UK National Hip Fracture Database indicate that each additional day of physiotherapy during the first postoperative week increases the odds of discharge (adjusted OR: 1.26, 95% CI: 1.19-1.33) [33,40]. Provision of 6-7 days of physiotherapy is associated with higher rates of discharge home, improved survival, superior mobility recovery, and lower 30-day readmission rates [40]. Modelling suggests that a hospital admitting 375 hip fracture patients annually could save 456 bed-days by implementing seven-day physiotherapy in the first postoperative week [33]. Additionally, a Norwegian observational study of 30,752 hip fractures found that comprehensive physiotherapy access improved health-related quality of life at four and 12 months post-fracture [41].

**Figure 2:**
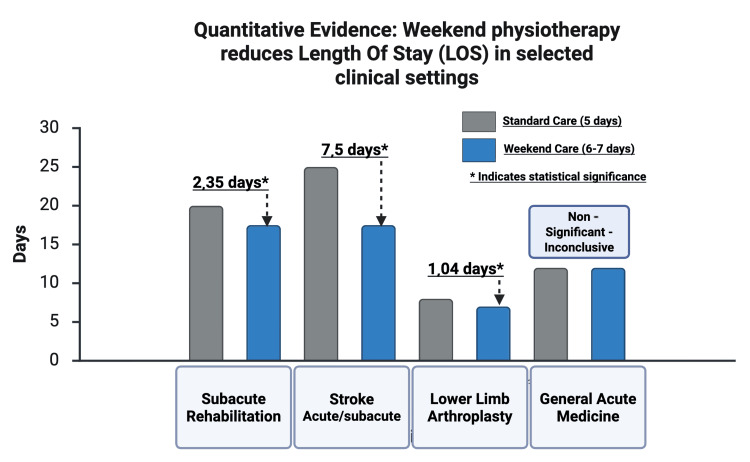
Quantitative evidence: the addition of weekend physiotherapy reduces hospital LOS in selected settings A grouped bar chart comparing hospital LOS in days for a five-day standard care model (grey bars) versus a comprehensive seven-/six-day model (blue bars) across different clinical specialties: subacute rehabilitation, acute neuro-acute (stroke unit), elective orthopaedic arthroplasty, and general acute medicine. Mean LOS reductions of ~2.35 days*, ~7.5 days*, and ~1.04 days* are shown for the extended model in subacute rehab, neuro-acute unit, and elective orthopaedic arthroplasty, respectively. LOS differences in general acute medicine were negligible and not statistically significant. Asterisks (*) indicate statistical significance. LOS: length of stay This figure was created using BioRender.

Evidence in elective hip and knee arthroplasty underscores the importance of early and continuous intervention. A comprehensive systematic review and meta-analysis found that adding weekend physiotherapy after elective lower limb arthroplasty significantly reduced LOS (WMD=-1.04 days) and improved functional outcomes [42]. Although a subsequent quasi-experimental study observed that acute LOS could appear longer in certain multivariable models, this occurred despite improved mobility and higher odds of direct discharge home [43]. Overall, the synthesised evidence supports that maintaining physiotherapy continuity over the weekend accelerates postoperative recovery in elective orthopaedic pathways [42,43] (see Figure 2).

Acute General Medical and Surgical Wards

The evidence for seven-day physiotherapy in acute general medical and surgical wards remains inconclusive. Two large stepped-wedge cluster RCTs involving 12 wards across two Australian hospitals (n=27,508 patients) found that removing weekend allied health services did not consistently worsen outcomes. In some analyses, the absence of weekend services was non-inferior or even superior to weekend provision [31]. Meta-analyses have similarly concluded that the clinical and cost-effectiveness of weekend allied health services in these acute settings remain unclear [3].

Intensive Care and Critical Illness

Direct comparative evidence in the ICU strongly supports continuous weekend rehabilitation. A service evaluation by Weblin et al. [44] compared mechanically ventilated patients during two periods, that is, a seven-day physiotherapy service and a restricted five-day service, with only emergency respiratory physiotherapy on weekends. The shift to a five-day model was associated with a delay in time to first mobilisation and significantly lower mobility scores at both ICU and hospital discharge. From a health systems perspective, the five-day service substantially increased the proportion of patients requiring ongoing downstream physiotherapy (either inpatient or home-based) from 49% to 81% [44]. These findings indicate that service gaps in the ICU impair acute functional trajectories and transfer the rehabilitation burden and costs to post-acute care settings.

Frequency-Outcome Relationships

A large retrospective cohort study of 243,779 patients across multiple diagnostic subgroups identified a clear dose-response relationship between physiotherapy visit frequency and functional outcomes [24]. Compared to two or fewer visits per week, the adjusted relative risk for functional improvement increased with higher frequency: 1.20 (95% CI: 1.14-1.26) for more than two to four visits, 1.42 (95% CI: 1.30-1.55) for more than four to seven visits, and 1.78 (95% CI: 1.55-2.03) for more than seven visits per week [24]. Similar graded associations were observed for discharge home and for the combined outcome of functional improvement plus discharge home [24].

Discussion

Summary of Principal Findings and Differential Effectiveness

This narrative review synthesises evidence comparing seven-day and five-day physiotherapy across hospital specialties. The findings indicate that extended physiotherapy services provide the greatest benefits in rehabilitation settings and post-hip fracture care, while evidence for acute general medical and surgical wards remains inconclusive [3,5,31-34,45]. In subacute rehabilitation and hip fracture pathways, patients are generally medically stable and possess clear functional goals, enabling physiotherapy to address deficits and facilitate discharge readiness. The dose-response relationship between therapy frequency and functional improvement is well established in these populations [21,24,25]. In intensive care, recent observational studies comparing seven-day and five-day rehabilitation services during successive COVID-19 waves found that the absence of weekend physiotherapy was associated with delayed mobilisation, reduced mobility at discharge, and increased need for ongoing rehabilitation, highlighting the vulnerability of this population to interruptions in physiotherapy provision [44]. Conversely, patients on acute medical and surgical wards often present with unstable conditions, competing diagnostic priorities, and shorter LOS, which limit physiotherapy's ability to influence outcomes significantly [2,31].

Mechanistic and Dose-Response Considerations

Neuroplasticity and motor learning frameworks provide strong support for increased therapy frequency in selected patient cohorts. Evidence from stroke physiotherapy trials demonstrates that greater amounts of motor training are associated with improved functional outcomes, with repetition density and temporal compression identified as key biological mechanisms driving neuroplastic adaptation [21-23]. However, the optimal therapy dose remains uncertain. Very high doses of early intervention, such as two to three times the usual care, have not consistently yielded additional benefits in randomised trials. This underscores the importance of balancing therapy intensity with patient tolerance and clinical context [22].

Health Economics and Resource Allocation

From a health economics perspective, the cost-effectiveness of seven-day physiotherapy is highly dependent on the clinical setting and the patient population. In subacute rehabilitation, reduced LOS combined with improved functional outcomes yields highly favourable cost-effectiveness ratios [38,39]. In hip fracture care, the potential for significant bed-day savings, estimated at 456 days annually per average hospital, provides a strong economic rationale for health systems [33]. In contrast, the lack of clear clinical benefit in acute general medical and surgical wards undermines the economic justification for universal extended services, indicating that resources should be allocated more strategically [3,31].

Implementation Considerations and Research Priorities

Effective implementation of seven-day physiotherapy requires addressing multiple contextual and operational challenges. Appropriate staffing models and skill mix are essential. Assigning experienced therapists to weekend shifts and minimising rotational staffing can help maintain therapeutic relationships, while delegating routine ambulation tasks to therapy assistants enables physiotherapists to focus on complex assessments [1,4,5,46].

Although increasing weekend physiotherapy staffing enhances the volume of mobility treatments and continuity of care, particularly in critical care, it also raises concerns about staff work-life balance and burnout. Addressing these concerns requires careful rotational scheduling and compensatory frameworks, such as time-off-in-lieu, to support workforce sustainability [47,48].

Strategic targeting is essential. Prioritising patients at high risk of rapid deconditioning or those with significant rehabilitation potential is likely to improve cost-effectiveness, rather than implementing universal coverage [1,2]. Weekend physiotherapy achieves the greatest benefit when it is fully integrated into multidisciplinary discharge planning, including coordination with families [4].

Equity and Sociological Considerations

**Figure 3:**
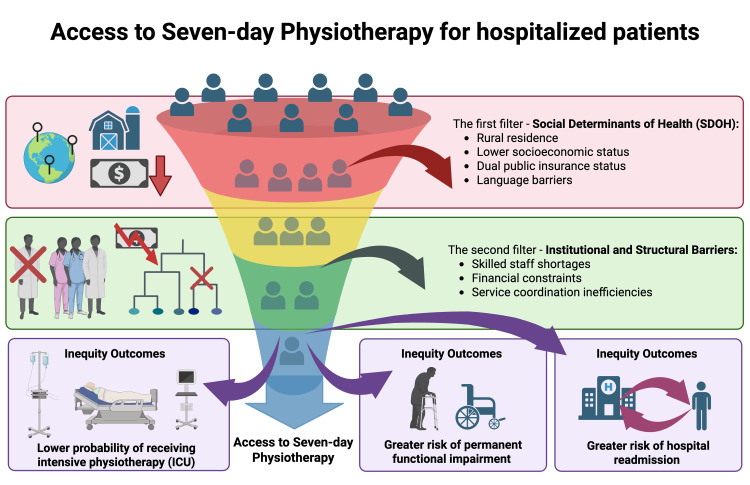
Sociological dimensions and equity: structural and sociological barriers influencing equitable access to intensive seven-day physiotherapy A conceptual model showing interconnected boxes of Filtros (cascadic barriers) leading to inequitable outcomes. Social determinants of health (SDOH): rural residence, lower socioeconomic status, dual insurance status (public/public), and language/linguistic barriers (Weblin et al. [44]). Institutional/structural barriers: skilled staff shortage, financial/budgetary constraints, and organisation/coordination inefficiencies (Sarkies et al. [3], Ottensmeyer et al. [48]). Arrows illustrate how these cumulative filters lead to the outcomes of resultados de inequidad (inequity outcomes): a lower probability of receiving intensive physiotherapy (e.g., ICU rehab), a greater risk of permanent functional impairment, and a greater risk of hospital readmission. This figure was created using BioRender.

Social determinants of health and structural inequities in access to inpatient rehabilitation are essential factors when interpreting and applying these findings. Large registry and administrative database studies demonstrate that older adults with dual insurance eligibility, rural residence, or limited language proficiency are less likely to receive intensive physiotherapy during hospitalisation, including in ICUs and rehabilitation units [28]. These inequities are reinforced at the institutional level. National surveys from Australia and Canada report substantial regional and sectoral disparities in weekend physiotherapy provision. Public, rural, and subacute facilities are significantly less likely to offer these services than private, metropolitan, and highly acute centres, primarily due to staffing shortages and financial constraints [48,49]. In the absence of explicit equity objectives, expanding seven-day services may preferentially benefit patients in already well-resourced hospitals, potentially exacerbating existing disparities in functional outcomes (see Figure 3).

Methodological heterogeneity across studies, including variations in intervention design, weekend service intensity, and outcome measurement, limits the ability to draw definitive conclusions about standardised service models [3,50,51]. The predominance of evidence from Australian and UK healthcare systems raises concerns about generalisability to other contexts with different financing structures, staffing models, and patient demographics [1,2]. For instance, hospitals operating within publicly integrated, universally funded models may more readily absorb the upfront weekend staffing costs to achieve long-term savings through reduced LOS. Conversely, in fragmented fee-for-service systems or low-resource settings, immediate budgetary constraints, workforce shortages, and variable insurance coverage present substantial barriers. In such environments, universal implementation is often unfeasible, making targeted, criteria-based pathways even more critical to maximise resource efficiency.

Future research should prioritise large multicentre trials with embedded economic evaluations that report incremental cost per QALY, particularly in acute stroke and hip fracture populations. Additionally, adopting standardised core outcome sets with 6-12‑month follow-up is necessary to assess the sustainability of gains [41,52].

This review has several limitations. As a narrative review rather than a full Preferred Reporting Items for Systematic Reviews and Meta-Analyses (PRISMA) systematic review, it may be subject to selection bias. Substantial clinical and methodological heterogeneity precluded meta-analysis across all settings. The evidence base is dominated by studies from Australia and the UK, which limits global generalisability. Publication bias is also possible, as trials demonstrating positive effects of weekend services may have been more likely to be published [3].

## Conclusions

Comprehensive seven-day physiotherapy services provide consistent clinical and economic benefits in subacute rehabilitation, acute stroke units, post-hip fracture pathways, and intensive care settings. In these targeted contexts, extended allied health coverage reduces hospital LOS, leads to meaningful functional improvements, and achieves favourable cost-effectiveness ratios. Therefore, current evidence supports prioritising the implementation and funding of seven-day models for these specific patient populations.

In contrast, for acute general medical and surgical wards, the evidence remains mixed and is insufficient to justify the routine, universal provision of weekend physiotherapy. Given constrained healthcare funding, administrators and clinicians should allocate weekend allied health resources to settings with the strongest evidence of benefit or to clearly defined high-risk patient subgroups with significant rehabilitation potential. Future research should emphasise large multicentre pragmatic trials with embedded economic evaluations, standardised core outcome measures, and extended follow-up. Additionally, implementation studies should explore contextual barriers and facilitators to sustainable seven-day services. Aligning service delivery models with this nuanced evidence base will enable health systems to maximise the value of physiotherapy while ensuring equitable and efficient use of limited clinical resources.
